# Identification of Gut Microbiome and Metabolites Associated with Acute Diarrhea in Cats

**DOI:** 10.1128/spectrum.00590-23

**Published:** 2023-07-10

**Authors:** Huasong Bai, Tong Liu, Songjun Wang, Wenhui Gong, Liya Shen, Song Zhang, Zhanzhong Wang

**Affiliations:** a Nourse Science Centre for Pet Nutrition, Wuhu, China; Texas A&M University

**Keywords:** domestic cat, QIIME2, gut microbiota, acute diarrhea, microbiome, metabolome

## Abstract

Changes in diet and environment can lead to acute diarrhea in companion animals, but the composition and interactions of the gut microbiome during acute diarrhea remain unclear. In this multicenter case-control study, we investigated the relationship between intestinal flora and acute diarrhea in two breeds of cats. Acutely diarrheic American Shorthair (MD, *n* = 12) and British Shorthair (BD, *n* = 12) and healthy American Shorthair (MH, *n* = 12) and British Shorthair (BH, *n* = 12) cats were recruited. Gut microbial 16S rRNA sequencing, metagenomic sequencing, and untargeted metabolomic analysis were performed. We observed significant differences in beta-diversity (Adonis, *P *< 0.05) across breeds and disease state cohorts. Profound differences in gut microbial structure and function were found between the two cat breeds. In comparison to healthy British Shorthair cats, *Prevotella*, *Providencia*, and *Sutterella* were enriched while *Blautia*, *Peptoclostridium*, and *Tyzzerella* were reduced in American Shorthair cats. In the case-control cohort, cats with acute diarrhea exhibited an increased abundance of *Bacteroidota*, *Prevotella*, and Prevotella copri and a decreased abundance of *Bacilli*, *Erysipelotrichales*, and *Erysipelatoclostridiaceae* (both MD and BD cats, *P *< 0.05). Metabolomic analysis identified significant changes in the BD intestine, affecting 45 metabolic pathways. Moreover, using a random forest classifier, we successfully predicted the occurrence of acute diarrhea with an area under the curve of 0.95. Our findings indicate a distinct gut microbiome profile that is associated with the presence of acute diarrhea in cats. However, further investigations using larger cohorts of cats with diverse conditions are required to validate and extend these findings.

**IMPORTANCE** Acute diarrhea is common in cats, and our understanding of the gut microbiome variations across breeds and disease states remains unclear. We investigated the gut microbiome of two cat breeds (British Shorthair and American Shorthair) with acute diarrhea. Our study revealed significant effects of breeds and disease states on the structure and function of the gut microbiota in cats. These findings emphasize the need to consider breed-related factors in animal nutrition and research models. Additionally, we observed an altered gut metabolome in cats with acute diarrhea, closely linked to changes in bacterial genera. We identified a panel of microbial biomarkers with high diagnostic accuracy for feline acute diarrhea. These findings provide novel insights into the diagnosis, classification, and treatment of feline gastrointestinal diseases.

## INTRODUCTION

The gastrointestinal (GI) tract of companion animals (feline and canine) harbors a complex microbial community, which includes bacteria, fungi, viruses, and protozoa (1), showing a high degree of phylogenetic diversity. Moreover, the gut microbiota is often considered an important “metabolic organ,” since it influences host health by catabolism, regulation of immunity, and defense against pathogen invasion (2). Changes in the gut microbiota which usually result from disease states are known as ecological dysbiosis (3). Molecular techniques such as 16S rRNA gene sequencing and DNA shotgun sequencing (metagenomics) have been used to characterize the gut microbiota of companion animals in different physiological states (4). In addition, a PCR-based dysbiosis index has been proposed to quantitatively assess gut dysbiosis in dogs (5). It is worth noting that a dysbiosis index for cats has also been proposed (6), which provides an important reference for further understanding the changes of the feline gut microbiome in different disease states.

Acute diarrhea in cats is a common GI disorder occurring at all ages, usually accompanied by nonpersistent diarrhea and GI symptoms for approximately 3 weeks (7), but its etiology is unknown. It is believed that this is the result of complex interactions between genetics, dietary factors, pathogen infection, antibiotic use, and the gut microbiota (8). While acute diarrhea is a distinct disease phenotype from chronic enteropathies such as inflammatory bowel disease (IBD) and small cell lymphoma (SCL), previous studies have shown that acute and chronic GI disorders alter the gut microbiota and impair host health in cats, as well as in many other mammalian hosts, including humans and canines (1, 9). Studies on dogs with GI disorders have revealed differences in gut microbiota diversity between those with acute diarrhea and IBD, but both conditions exhibited dysbiosis and fecal metabolite alterations (7, 10, 11). However, studies on the gut microbiota of cats affected by GI disorders are scarce, especially for acute diarrhea.

Previous studies have highlighted the occurrence of gut dysbiosis in cats with IBD and SCL, as evidenced by a decreased abundance of representatives of the phylum *Firmicutes* (*Rumenococcus* and *Turicibacteraceae*) and an increased abundance of representatives of the phylum *Enterobacteriaceae* (12). Notably, no significant differences were found between the gut microbiome of cats with IBD and SCL. Another study showed differences in the gut microbiota of cats in response to acute and chronic diarrhea (13). For instance, the abundance of *Bacteroidetes* was reduced in cats with chronic diarrhea, whereas that of *Erysipelobacter* and *Lactobacillus* was reduced in cats with acute diarrhea. However, although these studies enabled characterization of the gut microbiome of cats with acute or chronic diarrhea, breed differences were not taken into consideration. Currently, gut microbiome and metabolome integrated analysis has been widely used to understand the relationship between disease and gut microbiota (14). However, no studies have been conducted so far on GI disorders in cats, especially acute diarrhea.

Thus, the aim of the present study was to investigate changes in the gut microbiome of two cat breeds, i.e., American Shorthair and British Shorthair, showing symptoms of acute diarrhea, in comparison to healthy cats. Moreover, metagenomic and nontargeted metabolomic analyses were conducted on the feces of British Shorthair cats to explore the potential relationship between gut microbiota, metabolites, and acute diarrhea.

## RESULTS

### Clinical characteristics and demographics.

We recruited a total of 51 cats (consisting of American Shorthair and British Shorthair breeds) from six regions in China and collected fecal samples from each cat. The geographical locations of all recruited cats is given in Fig. S1 in the supplemental material. Three cats were excluded from the study: one due to prior antibiotic and medication use for encephalitis 7 days before the study and two due to internal parasite treatment 7 days before the study (Table S1). The remaining 48 cats were divided into four groups, i.e., healthy American Shorthair (MH; *n* = 12), healthy British Shorthair (BH; *n* = 12), acutely diarrheic American Shorthair (MD; *n* = 12), and acutely diarrheic British Shorthair (BD; *n* = 12), and underwent a case-control study (Fig. S2). We conducted statistical analysis of the clinical metadata for the study animals (Table S2). Overall, there were no significant differences among the four groups in terms of age (*P *= 0.746), gender (*P *= 0.696), body weight (*P *= 0.682), obesity status (*P *= 0.127), number of vaccinated (*P *= 1) or dewormed (*P *= 1) individuals, or location (*P *= 0.613), but there were significant differences in defecation frequency (*P *= 0.001). Additionally, fecal scores for healthy cats (MH, 2.33 ± 0.89; BH, 2.5 ± 1) and diarrheic cats (MD, 6.17 ± 0.72; BD, 6.17 ± 0.83) were significantly different. The duration of diarrhea was 6.6 ± 2.2 days for the MD group and 7 ± 2.3 days for the BD group. Finally, there were no significant differences in macronutrient composition of commercial feed used in the four sample groups, including protein (*P *= 0.680), fat (*P *= 0.374), and carbohydrate (*P *= 0.366).

### Changes in gut microbiota diversity in cats of different breeds and disease status.

**FIG 1:**
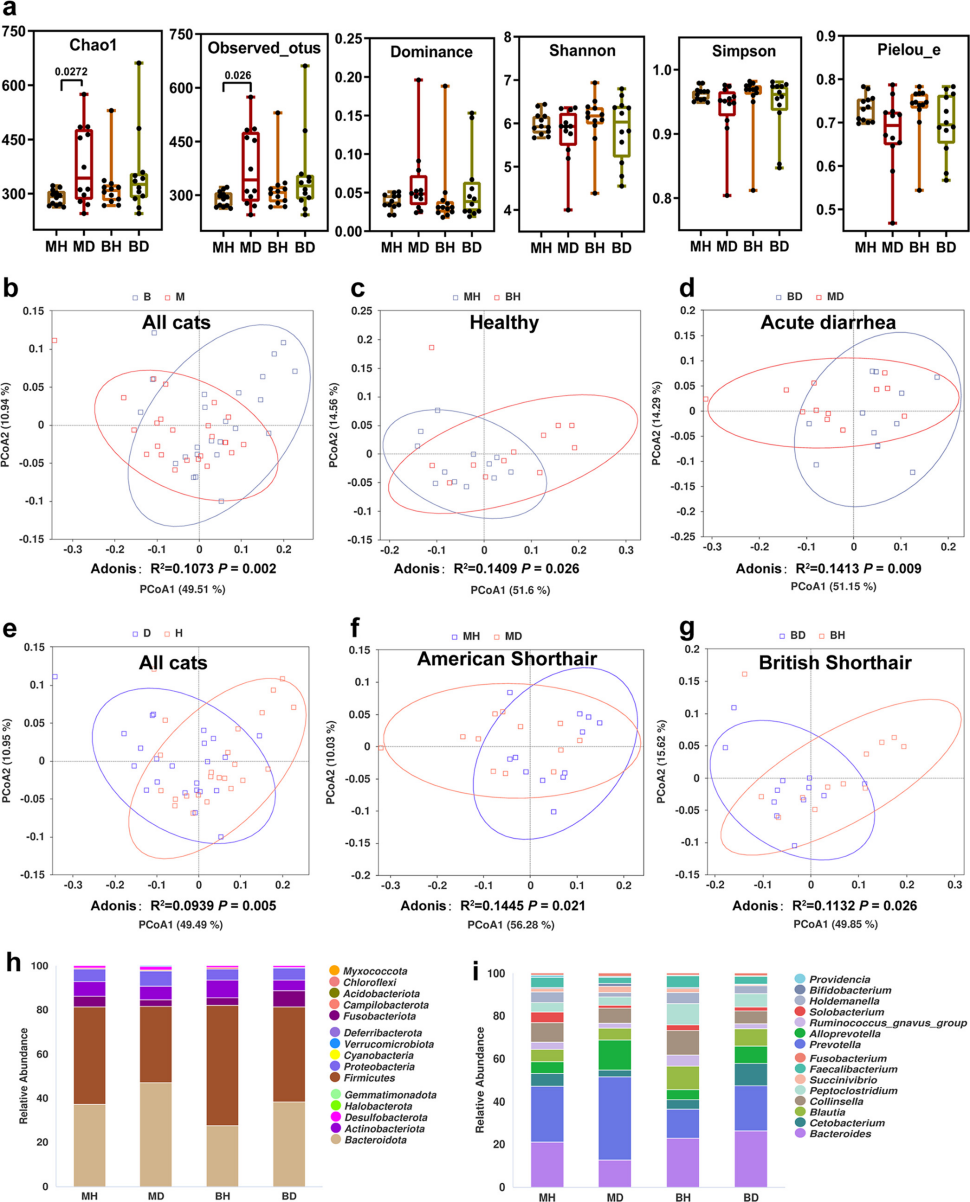
Diversity analysis and species distribution stacking plots between groups under different cohorts. (a) Alpha diversity between healthy and acute diarrhea groups from American Shorthair (MH and MD, respectively) and British Shorthair (BH and BD, respectively) cats. A *P* value of <0.05 was considered significant (using Wilcoxon rank sum test), and no labels indicate no significant differences. (b to g) Beta diversity analysis of different cohorts using PCoA based on the weighted UniFrac distance algorithm. The horizontal and vertical axes represent the first two principal components, and the percentages indicate the contribution of each principal component to the variation between the samples. Each dot in the plot represents a sample, with samples from the same group being represented in the same color. Significance between groups was assessed using permutation multivariate analysis of variance (Adonis) test, with *R*^2^ representing the proportion of variation explained by different grouping factors. An *R*^2^ of >0.05 indicates differences between groups, and a *P* of <0.05 indicates statistically significant differences between groups. (h to i) Cumulative bar chart of species relative abundance, with the top 15 species ranked in terms of maximum abundance at each phylum level (h) and genus level (i) according to different groupings.

To assess the richness and diversity of microbial communities, we performed alpha-diversity and beta-diversity analysis on different groups. Overall, alpha-diversity indexes differed between MH and MD, with Chao 1 (*P *= 0.027) and observed operational taxonomic unit (OTU) (*P *= 0.026) indexes significantly higher in MD, indicating a greater abundance in the gut microbiota of cats in the MD group (Fig. 1a). However, dominance, Shannon, Simpson, and Pielou indexes did not significantly differ between MH and MD. In addition, observed OTU, Chao1, dominance, Shannon, Simpson, and Pielou indexes did not remarkably differ when disease-control sample groups of British Shorthair cats (BH and BD) or the two cat breeds (MH and BH) were compared. Moreover, age and gender did not significantly correlate with changes in any of the analyzed alpha-diversity indexes (*P *> 0.05).

We evaluated beta-diversity metrics for breeds and disease status to determine whether subgroups were biologically different. Principal-coordinate analysis (PCoA) was conducted on different subgroups, and the significance of differences was tested using Adonis nonparametric multivariate analysis of variance. Significant differences were found in the diversity of the gut microbial community based on breed, whether considering the cohort of all cats (Fig. 1b, *P *= 0.002), healthy cats (Fig. 1c, *P *= 0.026), or cats with acute diarrhea (Fig. 1d, *P *= 0.009). This difference was significant between the MH and BH groups (*P *= 0.006) after subsequent correction for the effects of age and gender using multivariate analysis by linear models (MaAsLin). When based on disease status (Fig. 1e to g), microbial community diversity significantly differed between cats with acute diarrhea and healthy cats, when considering the cohort of all cats (*P *= 0.005) or just American Shorthair (*P *= 0.021) or British Shorthair (*P *= 0.026). After adjustment for gender and age, differences between cats in the acute diarrhea group and healthy group were still significant, when considering the cohort of all cats (*P *= 0.001) or just American Shorthair (*P *= 0.007) or British Shorthair (*P *= 0.013).

### Differences of microbial communities in different breeds and diseases.

The gut bacterial community of cats was dominated mainly by *Bacteroidota* (27.6 to 38.4%), *Firmicutes* (34.6 to 54.4%), *Fusobacteriota* (2.9 to 7.3%), *Actinobacteriota* (4.7 to 8.1%), and *Proteobacteria* (4.9 to 7.0%) in the four subgroups (Fig. 1h). This is consistent with previous reports (1). At the genus level, the distribution of gut bacterial communities in cats was primarily *Bacteroides* (8.5 to 16.5%), *Prevotella* (8.4 to 26.2%), *Cetobacterium* (2.0 to 6.5%), *Alloprevotella* (2.8 to 9.4%), *Blautia* (3.7 to 6.8%), *Ruminococcusgnavus* group (1.4 to 3.1%), and *Collinsella* (3.8 to 7.1%) in the four subgroups (Fig. 1i). Overall, it can be stated that the structure of microbial communities in cats changed based on breed or disease status.

**FIG 2:**
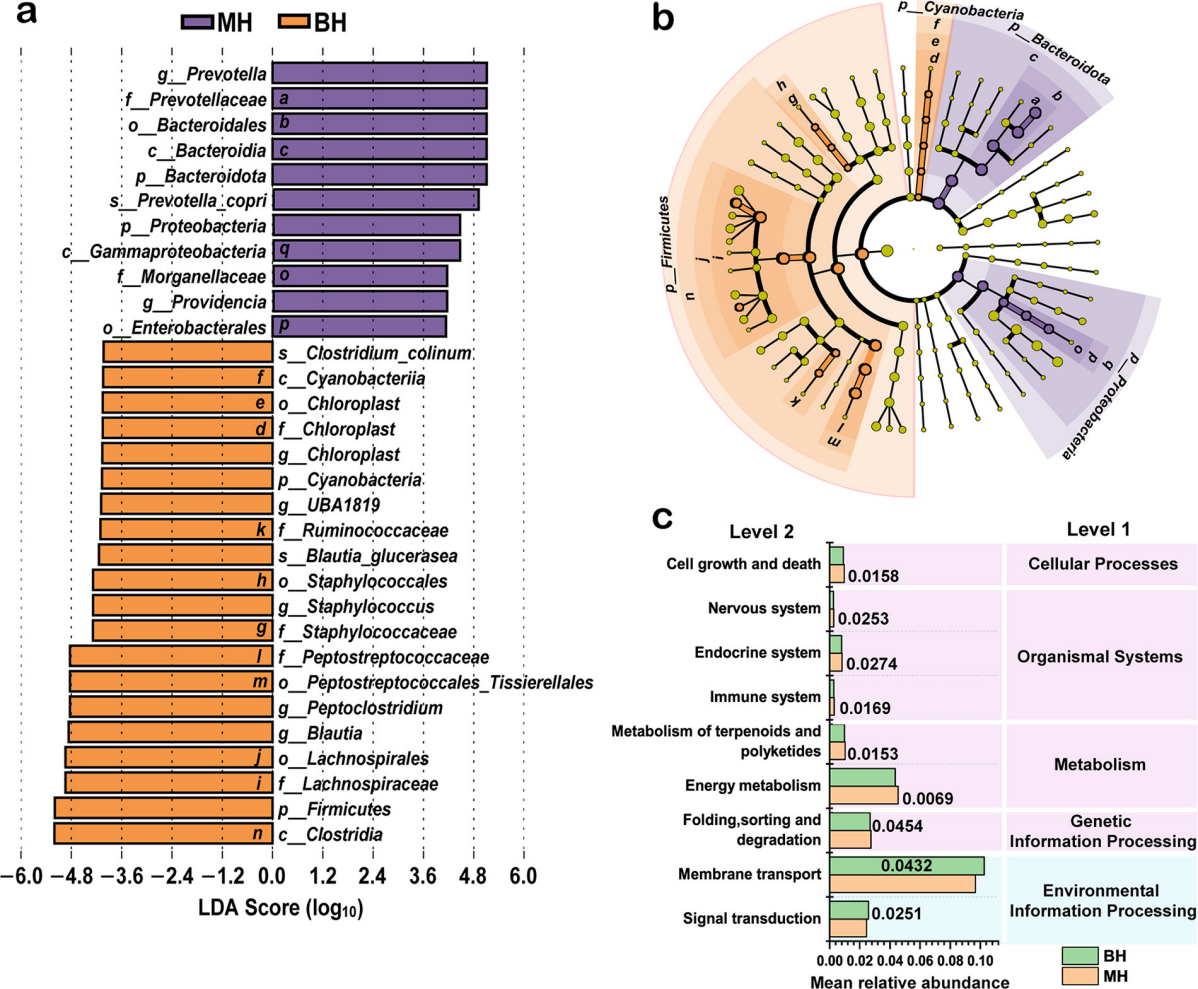
Differential bacterial species and microbial community function predictions between healthy American Shorthair (MH) and British Shorthair (BH) cats. (a) Histogram of the distribution of LDA values between MH and BH using LEfSe; species with LDA scores of >4 were considered to be significantly different in abundance between groups. (b) Evolutionary branching diagram between MH and BH. Circles radiating from inside to outside represent taxonomic levels from clade to species, respectively. Nodes represent a taxon at that level, and their size is proportional to the relative abundance. Labeled letters and differential species colors correspond to those in panel a. Yellow nodes are species with no significant differences. (c) Histogram of the relative abundance of functions between MH and BH based on Tax4Fun at the second annotation level, with a *P* of <0.05 considered significant (*t* test).

To further identify differential species at different taxonomic levels, Metastats analyses were conducted after correction for gender and age. As shown in Table S3, considering cat breeds, at the phylum level, the abundances of *Bacteroidota* (53.1 ± 4.1% versus 31.3 ± 6.3%, *P *= 0.012) and *Proteobacteria* (11.2 ± 1.5% versus 6.0 ± 0.8%, *P *= 0.004) were significantly higher in MH than in BH, whereas the abundance of *Firmicutes* (35.0 ± 3.8% versus 62.0 ± 6.7%, *P *= 0.003) was significantly lower in MH. At the genus level, *Prevotella* (52.6 ± 4.1% versus 30.5 ± 6.2%, *P *= 0.006), *Providencia* (2.1 ± 1.5% versus 0.2 ± 0.1%, *P *= 0.021), and *Sutterella* (9.0 ± 1.2% versus 5.7 ± 0.8%, *P *= 0.046) were the dominant genera in MH, while *Blautia* (13.3 ± 1.8% versus 25.4 ± 4.2%, *P *= 0.011), *Peptoclostridium* (10.0 ± 1.4% versus 22.5 ± 3.1%, *P *= 0.002), and *Tyzzerella* (0.8 ± 0.1% versus 1.7 ± 0.5%, *P *= 0.034) were significantly lower than in BH. Moreover, linear discriminant analysis (LDA) with effect size (LEfSe) was performed to identify differentially enriched representative microbial clusters with LDA scores of >4, which would enable distinguishing between MH and BH. LEfSe (Fig. 2a) enabled the identification of differentially abundant bacterial taxa in MH and BH. Four bacterial phyla enabled the discrimination between MH and BH (Fig. 2b), with *Bacteroidota* (LDA = 5.108, *P *= 0.015) and *Proteobacteria* (LDA = 4.449, *P *= 0.015) more abundant in MH, while *Firmicutes* (LDA = 5.193, *P *= 0.004) and *Cyanobacteria* (LDA = 4.067, *P *= 0.0231) were enriched in BH.

In cohorts with acute diarrhea, Metastats analysis (Table S3) revealed that the abundance of *Bacteroidota* (85.4 ± 3.6% versus 74.0 ± 3.0%, *P *= 0.030) was significantly higher in MD, which included *Prevotella* (59.5 ± 5.3% versus 39.7 ± 4.2%, *P *= 0.015) and *Muribaculaceae* (1.2 ± 0.3% versus 0.3 ± 0.1%, *P *= 0.009) within this phylum. Conversely, the abundance of *Firmicutes* (10.6 ± 3.0% versus 22.6 ± 2.9%, *P *= 0.007) was significantly lower in MD, with significantly lower levels of genera such as *Solobacterium* (2.3 ± 0.9% versus 8.1 ± 2.7%, *P *= 0.030), *Catenibacterium* (1.4 ± 0.4% versus 4.4 ± 1.0%, *P *= 0.007), and *Tyzzerella* (0.2 ± 0.1% versus 0.5 ± 0.1%, *P *= 0.006), among others, than in MH. In addition, *Bacteroidota* (67.0 ± 3.4% versus 44.6 ± 7.8%, *P *= 0.015) and *Prevotella* (64.4 ± 3.2% versus 43.2 ± 7.8%, *P *= 0.022) were significantly higher in BD than in BH, while *Actinobacteriota* (20.3 ± 2.5% versus 43.6 ± 6.9%, *P *= 0.009), *Collinsella* (19.8 ± 2.4% versus 42.8 ± 6.8%, *P *= 0.005), and *Erysipelatoclostridium* (0.4 ± 0.1% versus 1.1 ± 0.3%, *P *= 0.016) were significantly lower.

**FIG 3:**
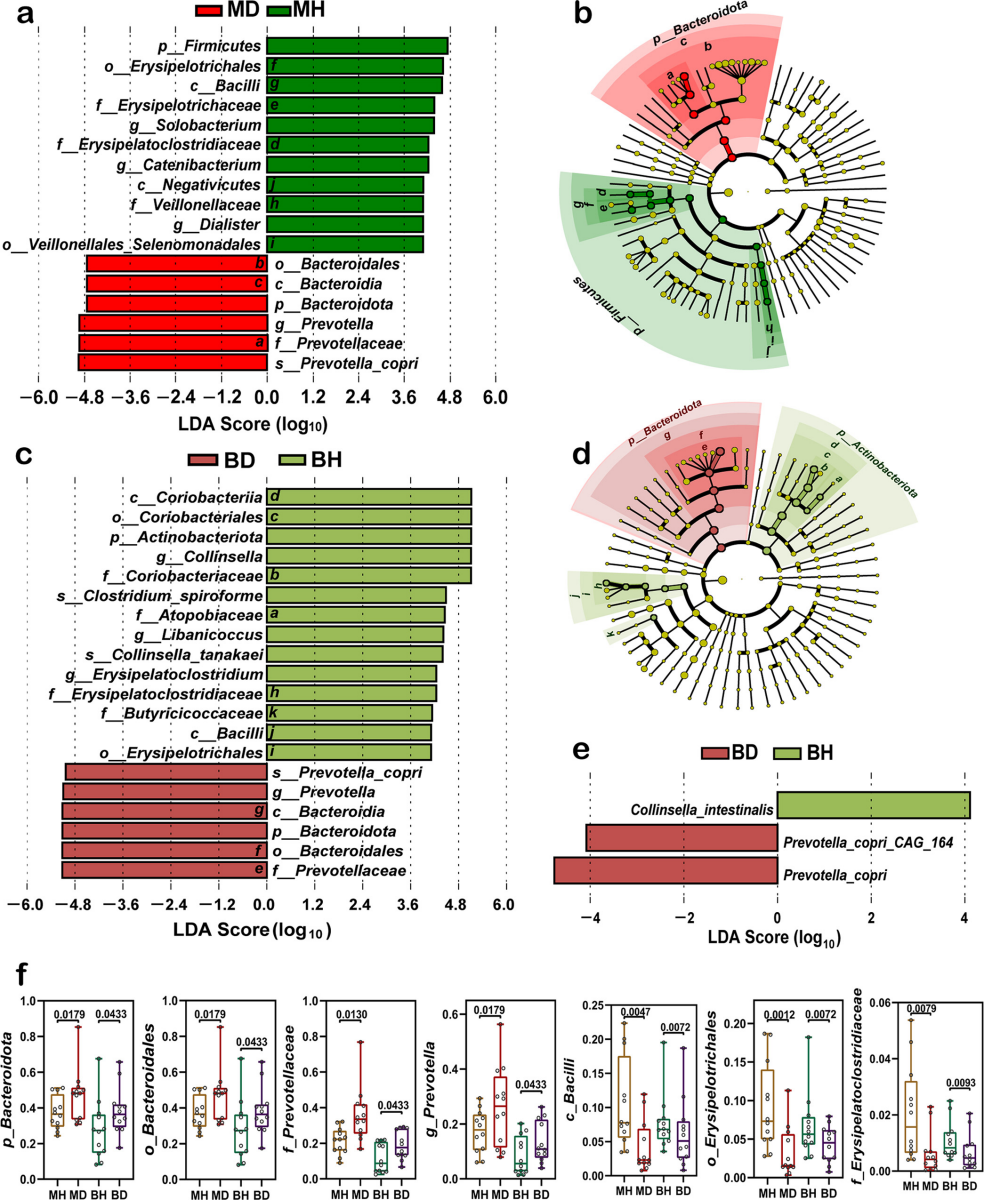
Species analysis of differences based on breeds between healthy and diarrheic cats using LEfSe (LDA score > 4). (a and b) Histogram of the distribution of LDA values and evolutionary branching between MH and MD, with the letters and colors labeled in panel b corresponding to those in panel a. (c and d) Histogram of the distribution of LDA values and evolutionary branching between BH and BD, with letters and colors in panel d corresponding to those in panel c. All yellow nodes are species with no significant differences. (e) Histogram of LDA value distribution between BH and BD based on metagenomics. (f) Box plots of species differences between groups; these taxa appeared together in BD and MD. A *P* of <0.05 was considered a significant difference (Metastats analysis).

LEfSe was used to compare healthy and diarrheic cats within each breed to enable distinguishing signature clusters. In American Shorthair cats, three genera (*Dialister*, *Catenibacterium*, and *Solobacterium*) were enriched in the healthy group, and one genus (*Prevotella*) and one species (Prevotella copri) were enriched in the acute diarrhea group (Fig. 3a). Among British Shorthair cats, three genera (*Libanicoccus*, *Collinsella*, and *Erysipelatoclostridium*) and two species (Clostridium spiroforme and Collinsella tanakaei) were enriched in the healthy group, while one genus (*Prevotella*) and one species (*P. copri*) were enriched in the acute diarrhea group (Fig. 3c). Moreover, LEfSe of metagenome sequencing (Fig. 3e) showed that *C. tanakaei* was enriched in the BH group, while *P. copri* and *P. copri* CAG164 were enriched in the BD group, which is consistent with the results of 16S rRNA gene sequencing. Interestingly, we found that differentially enriched signature groups in diarrheic cats were *Bacteroidota*, *Bacteroidales*, *Prevotellaceae*, *Prevotella*, and *P. copri*, which were commonly found in the gut microbiota of American Shorthair and British Shorthair cats (Fig. 3b and d). Furthermore, the class *Bacilli* within the phylum *Firmicutes* and the family *Erysipelatoclostridiaceae* within the order *Erysipelotrichales* were the abundant signature communities that were differentially found in healthy American Shorthair and British Shorthair cats (Fig. 3f).

### Prediction of microbial community function.

**FIG 4:**
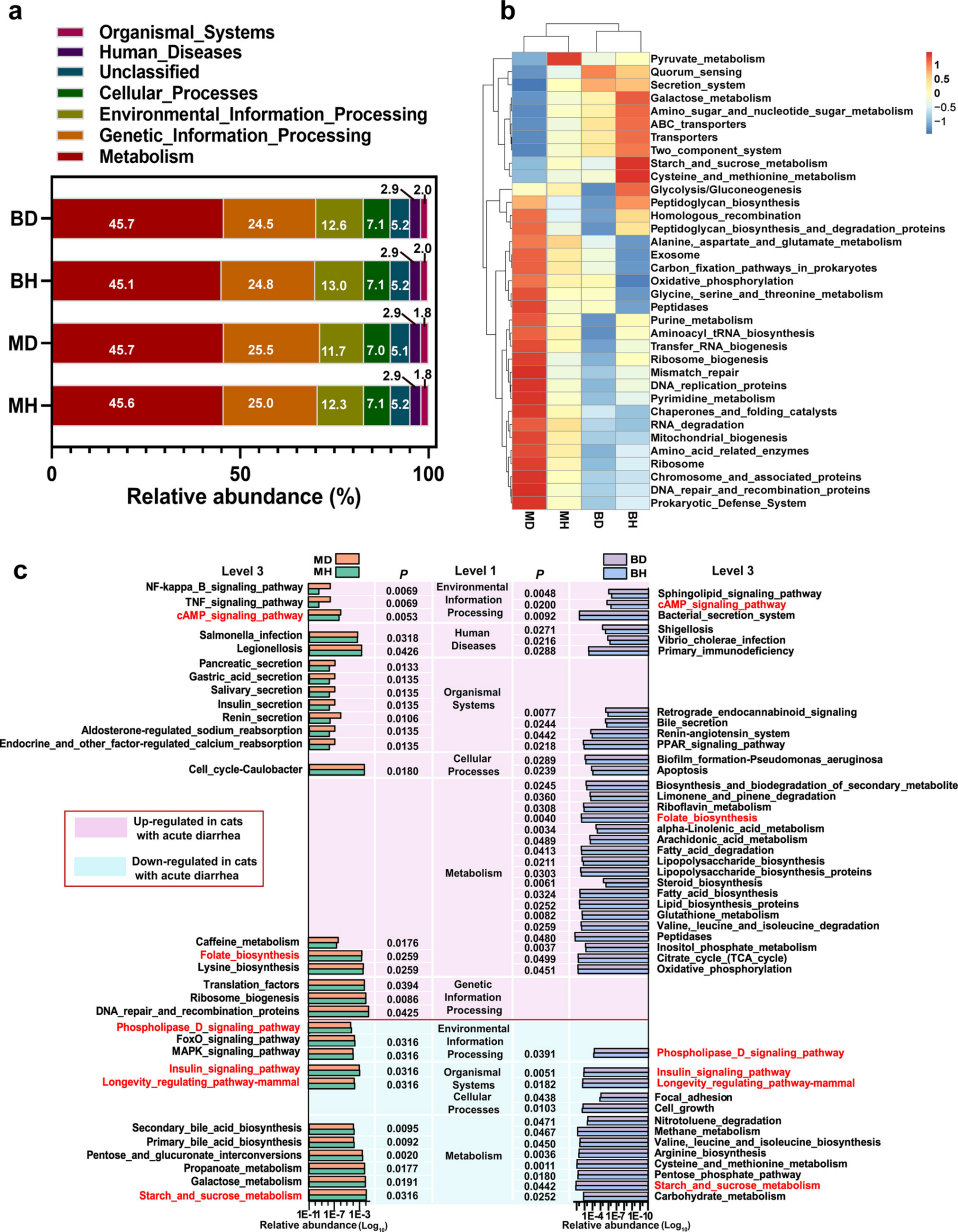
Functional annotation and variance analysis of the gut microbiota of healthy cats and cats with acute diarrhea from two different breeds based on Tax4Fun. (a) Bar stack plot of the top 10 relative functional abundances of different groupings at the first annotation level. (b) Clustering heat map for each grouping, by selection of the top 35 functions in the third annotation level in terms of abundance. Horizontal is the grouping, vertical is the functional annotation information, and left is the functional clustering tree. (c) Histogram of relative abundance of differential flora functions in healthy cats and cats with acute diarrhea of different breeds. A *P* of <0.05 was considered a significant difference (*t* test). Functional pathways shared by the two breeds of cats with acute diarrhea are marked in red.

To analyze the differences of the function of the gut microbiota of healthy and diarrheic cats based on breeds, we performed Tax4Fun functional prediction analysis based on the nearest neighbor method with minimal 16S rRNA gene sequence similarity. From the functional information pileup plot (Fig. 4a) at the first annotation level, the main functions of the gut bacterial community in the four sample groups were metabolism (45.1 to 45.7%), genetic information processing (24.5 to 25.5%), environmental information processing (11.6 to 13.0%), cellular processes (7.0 to 7.1%), unclassified (5.0 to 5.2%), human diseases (2.9%), and organismal systems (1.8 to 2.0%). The top 35 most enriched functions in each sample group at the third annotation level were selected for clustering to generate heat maps. The analysis illustrated that functions of the gut microbial community in healthy and diarrheic cats were clearly divergent between cat breeds, suggesting a change in microbial community functions based on breed and disease state (Fig. 4b).

To further investigate the differences in microbial community function between sample groups, *t* tests were conducted on the annotated microbial functions at different levels. We observed that the most abundant functions in BH were signal transduction (*P *= 0.025) and membrane transport (*P *= 0.043), which suggests that British Shorthair cats have an advantage over American Shorthair cats in environmental information processing of the gut microbiota (Fig. 2c). In contrast, folding, sorting, and degradation (*P *= 0.045), energy metabolism (*P *= 0.006), metabolism of terpenoids and polyketides (*P *= 0.015), immune system (*P *= 0.016), endocrine system (*P *= 0.027), nervous system (*P *= 0.025), and cell growth and death (*P *= 0.015) were more abundant in BH, which suggests that the gut microbiota of American Shorthair cats is more functionally enriched in processes related to genetic information processing, metabolism, organismal systems, and cellular processes than that of British Shorthair cats. In addition, microbiota function was altered between healthy cats and diarrheic cats of both breeds (Fig. 4c). Among these, starch and sucrose metabolism, longevity regulating pathway–mammal, insulin signaling pathway, and phospholipase D signaling pathway were significantly reduced in diarrheic cats (*P *< 0.05), and folate biosynthesis and cAMP signaling pathway were significantly increased (*P *< 0.05), in comparison with American Shorthair or British Shorthair healthy cats. Furthermore, in separate cohorts of American Shorthair cats, galactose metabolism, propanoate metabolism, pentose and glucuronate interconversions, primary bile acid biosynthesis, secondary bile acid biosynthesis, mitogen-activated protein kinase (MAPK) signaling pathway, and FoxO signaling pathway were significantly reduced (*P *< 0.05) in MD compared with MH; in contrast, DNA repair and recombination proteins, ribosome biogenesis, translation factors, lysine biosynthesis, folate biosynthesis, caffeine metabolism, cell cycle caulobacter, legionellosis, Salmonella infection, insulin secretion, pancreatic secretion, tumor necrosis factor (TNF) signaling pathway, NF-kappa B signaling pathway, and other functions were significantly increased in MD (*P *< 0.05). In British Shorthair cohorts, carbohydrate metabolism, pentose phosphate pathway, cysteine and methionine metabolism, arginine biosynthesis, valine, leucine, and isoleucine biosynthesis, methane metabolism, nitrotoluene degradation, cell growth, and focal adhesion were significantly decreased (*P *< 0.05) in BD compared with that in BH; conversely, oxidative phosphorylation, tricarboxylic acid cycle, inositol phosphate metabolism, peptidases, valine, leucine, and isoleucine degradation, glutathione metabolism, lipid biosynthesis proteins, fatty acid biosynthesis, steroid biosynthesis, lipopolysaccharide biosynthesis proteins, lipopolysaccharide biosynthesis, fatty acid degradation, arachidonic acid metabolism, apoptosis, retrograde endocannabinoid signaling, primary immunodeficiency, shigellosis, and bacterial secretion system were significantly increased in BD (*P *< 0.05).

Moreover, it was observed that a reduction in carbohydrate-related metabolic capacity was a common feature of acute diarrhea in the two cat breeds evaluated here. To this end, significantly differential carbohydrases and orthologous gene clusters between BH and BD were further explored using Metastat analysis in accordance with functional annotation results of metagenome sequencing related to CAZy and eggNOG databases in British Shorthair cats. The results (Fig. S3) revealed that the abundance of enzymes such as 6-phospho-beta-glucosidase (EC 3.2.1.86), beta-d-fucosidase (EC 3.2.1.38), and ABA-specific beta-glucosidase (EC 3.2.1.175) was significantly decreased in BD (*P *< 0.05), while the abundance of enzymes such as alpha-l-arabinofuranosidase (EC 3.2.1.55), beta-xylosidase (EC 3.2.1.37), and alpha-glucosidase (EC 3.2.1.20) was significantly increased in BD (*P*< 0.05); collectively, these enzymes likely play a key role in carbohydrate catabolism and metabolism. In addition, a screening was conducted to identify signature proteins associated with acute diarrhea in cats by comparative analysis of orthologous gene clusters. The abundance of orthologous clusters such as binding to the 23S rRNA (by similarity), 4Fe-4S ferredoxin iron-sulfur binding domain protein, and pyruvate kinase was significantly lower in the BD group than in the BH group (*P *< 0.05), while the abundance of transporter, transposase (IS4 family), and glycosyl transferase (group 1) was significantly increased in the BD group (*P *< 0.05) (Fig. S4).

### Random forest models for the diagnosis of acute diarrhea in cats.

**FIG 5:**
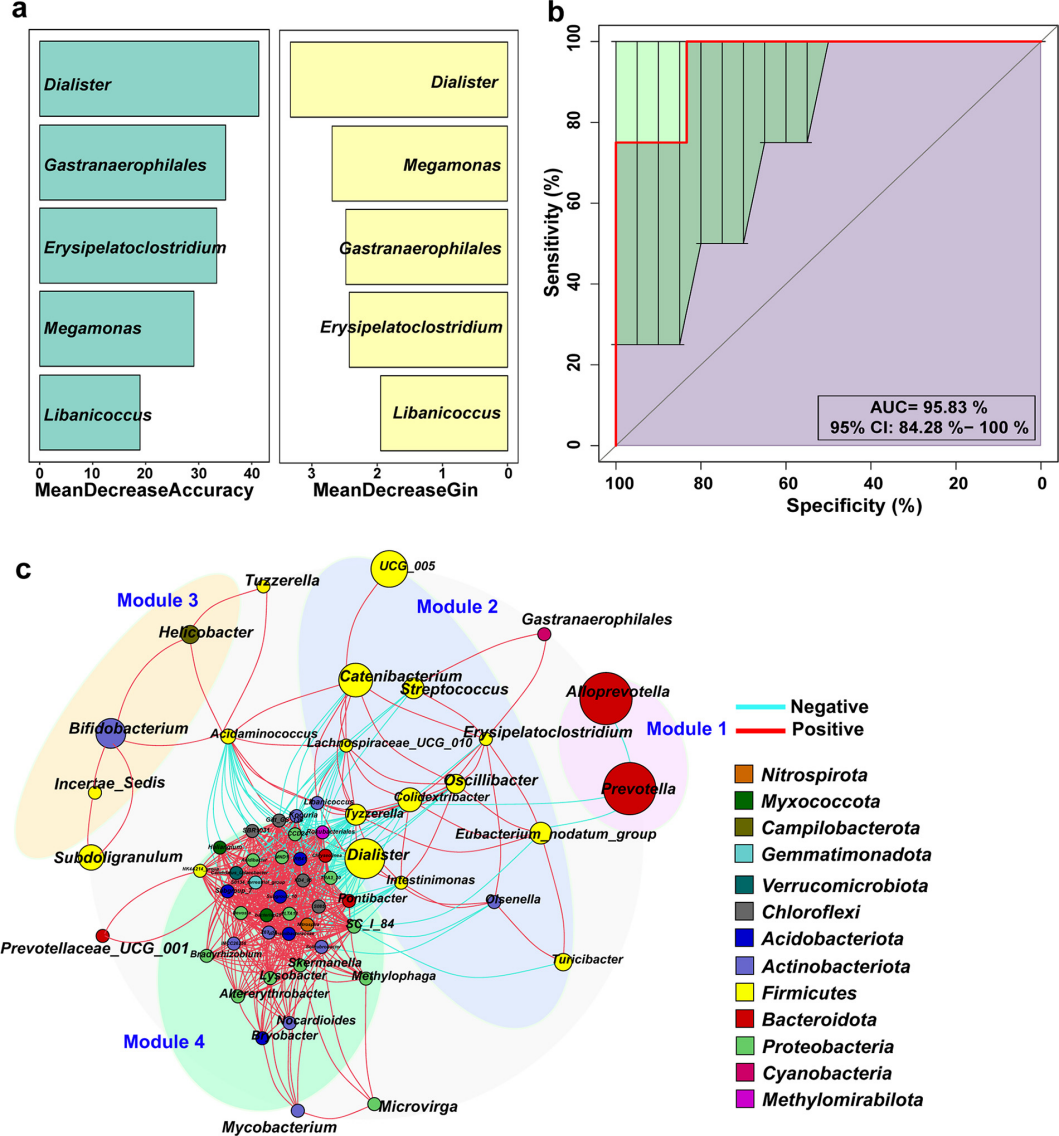
Diagnostic model for acute diarrhea in cats based on a random forest model using fecal microbiome data sets. (a) Variable importance ranking plots for five genera constructed by random forest analysis for predicting acute diarrhea in cats. The horizontal coordinates are the mean decrease accuracy and mean decrease gin indexes, respectively, with larger values indicating a greater importance of the genus. (b) ROC curve of the random forest prediction model for the five genera, showing excellent diagnostic performance for acute diarrhea in cats, with an area under the ROC (AUC) of 95.83%. (c) Cooccurrence network diagram of the gut microbiome of cats with acute diarrhea based on the Spearman correlation matrix. The nodes represent different genera, and their sizes indicate the average relative abundances. The colors of the nodes indicate the phyla to which they belong. The color of the linkage between nodes represents correlation, with red indicating a positive correlation and blue indicating a negative correlation.

Subsequently, a diagnostic model for cats with acute diarrhea was constructed based on a random forest algorithm. Sample groups of American and British Shorthair cats were divided into a healthy group (*n* = 24) and an acute diarrhea group (*n* = 24) to obtain a more broadly representative mixed clinical cohort. Information related to gender, age, and breed were included as covariates to eliminate interference onto the predictive model using multiple linear regression analysis. Subsequently, after the cohort (*n* = 48) was split into independent training and validation groups, a random forest-based machine learning model was used to select the clusters of genera that most effectively enabled the differentiation of cats with acute diarrhea from healthy cats. The average cross-validation area under the receiver operating characteristic (ROC) curve (AUC) of the random forest model after seven random splits in the training data set was 0.80 (standard deviation [SD] = 0.01) (Fig. S5a). In the holdout validation set, the AUC was 0.89 (SD = 0.11) (Fig. S5b). In addition, five genera, i.e., *Dialister*, *Megamonas*, *Gastranaerophilales*, *Erysipelatoclostridium*, and *Libanicoccus*, were identified (Fig. 5a), and the AUC was 0.95 for the randomly generated validation set (Fig. 5b). Of note, further research is necessary to validate and refine the diagnostic model using larger and more diverse cohorts.

### Cooccurrence of microbial communities in cats with acute diarrhea.

In order to explore the cooccurrence relationship of species groups in the gut of cats with acute diarrhea, a species correlation coefficient matrix was obtained based on Spearman correlation analysis. The cooccurrence network diagram (Fig. 5c) showed four modular species groups in the gut microbiota of cats with acute diarrhea. Among these, module 2 represented by representatives of the phylum *Firmicutes* (*Dialister*, *Catenibacterium*, *Colidextribacter*, Eubacterium nodatum group, *Tyzzerella*, and *Oscillibacter*, among others) was the dominant community in diarrheic cats. Moreover, a clear negative correlation was found between module 2 and module 1 represented by representatives of the phylum *Bacteroidota* (*Prevotella* and *Alloprevotella*). Module 3 was dominated by *Bifidobacterium* and positively interacted with module 2. Module 4 was composed mainly of representatives of the phyla *Acidobacteriota*, *Proteobacteria*, and *Campylobacterota*, among others, and the interaction within the species group was close, but a negative correlation trend was found with module 2.

### Fecal nontargeted metabolomics.

**FIG 6:**
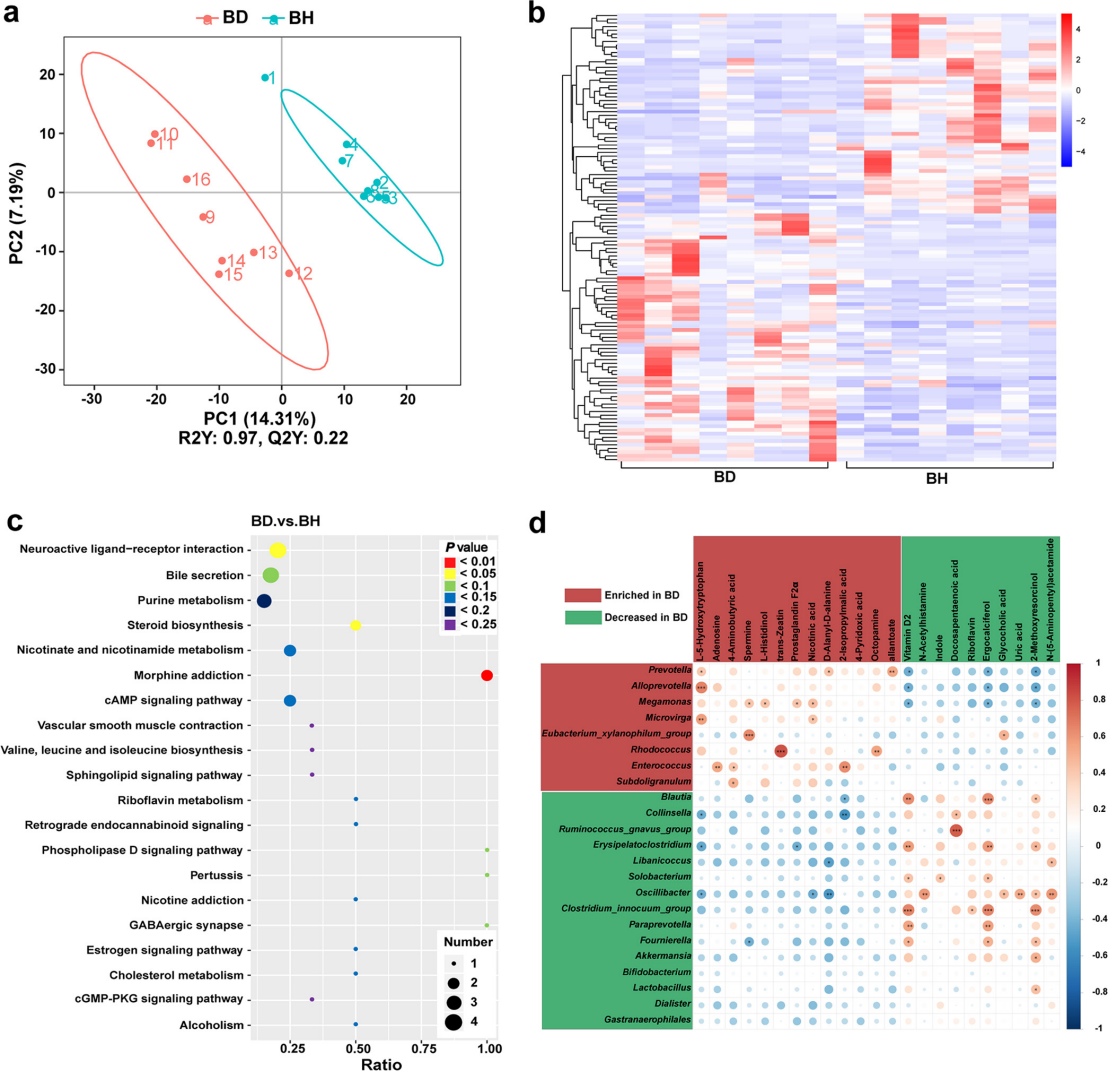
Fecal nontargeted metabolomics of healthy British shorthair cats (BH) and British Shorthair cats with acute diarrhea (BD). (a) PLS-DA of fecal nontarget metabolites for BH and BD. R2Y, represents the goodness of fit of grouping model. Q2Y, represents the predictive ability of grouping model. R2Y and Q2Y are both >0, indicating that the grouping model is stable and reliable. (b) Clustered heat map of the differential metabolites of BH and BD. Horizontal is the grouping. Vertical is the clustering of metabolites, with shorter clustering branches representing higher similarity. (c) Bubble plots of the KEGG pathway enriched for differential metabolites of BH and BD. The horizontal coordinate is the ratio of the number of differential metabolites in that metabolic pathway to the number of total metabolites identified in that pathway. The color of the dots represents the *P* value (hypergeometric test). The size of the dots represents the number of differential metabolites in the pathway. (d) Pearson correlation analysis of differential genera with differential metabolites for BD and BH. The size of the dots indicates the Pearson correlation coefficient (|rho|). The color of the dots indicates correlation, with blue indicating negative correlation and red indicating positive correlation. *, *P *≤ 0.05; **, *P *≤ 0.01; ***, *P *≤ 0.001.

Additionally, nontargeted metabolomic analysis was conducted on BH and BD fecal samples to further explore metabolic changes in cats with acute diarrhea. A total of 1,458 metabolites were identified from fecal samples; among these, a total of 121 metabolites were considered significantly differential, of which 67 were upregulated in BD and 54 metabolites were downregulated in BD (Table S4). Notably, l-5-hydroxytryptophan, 4-aminobutyric acid, adenosine, spermine, and nicotinic acid were dramatically upregulated in BD; in contrast, the levels of vitamin D_2_, ergocalciferol docosapentaenoic acid, indole, and 2-methoxyresorcinol were significantly reduced in BD. Moreover, partial least-squares discriminant analysis (PLS-DA) analysis (Fig. 6a) showed a significant distinction in metabolic profiles between BH and BD. Hierarchical clustering analysis of the differential metabolites (Fig. 6b) revealed distinctly different metabolic profiles between BH and BD, suggesting altered metabolic function in cats with acute diarrhea.

To further determine the biological functions of the identified differential metabolites, pathway annotation and KEGG enrichment analysis were conducted. The differential metabolites were found enriched in 45 metabolic pathways (Table S5), and Fig. 6c depicts a bubble map of the top 20 most enriched pathways. Overall, 13 enriched pathways were associated with abnormal 4-aminobutyric acid metabolism, such as morphine addiction pathway, neuroactive ligand-receptor interaction, GABAergic pertussis (GABA, γ-aminobutyric acid), synapse, and cAMP signaling pathway, among others. Moreover, 15 pathways were associated with abnormal lipid or steroid hormone metabolism, including steroid biosynthesis, bile secretion, phospholipase D signaling pathway, and cholesterol metabolism, among others. Furthermore, 12 pathways were associated with abnormal amino acid metabolism, which included valine, leucine, and isoleucine biosynthesis, histidine metabolism, phenylalanine, tyrosine and tryptophan biosynthesis, alanine, aspartate, and glutamate metabolism, and others. Finally, four pathways were associated with abnormal vitamin or cofactor metabolism, such as nicotinate and nicotinamide metabolism and riboflavin metabolism.

### Integrated analysis of 16S rRNA sequencing and nontargeted metabolomics.

It can be hypothesized that alterations in the gut metabolome of cats with acute diarrhea are likely related to perturbations in the dynamic microbiome. To further explore the relationship between the gut microbiome and the metabolome, Pearson correlation analysis was conducted for differential gut microbes and metabolites. We found that the genera exhibiting increased abundance in BD showed a positive correlation with significantly upregulated metabolites in BD and a negative correlation with downregulated metabolites and vice versa (Fig. 6d). Notably, among genera that were increased in BD, *Prevotella* showed positive correlations with l-5-hydroxytryptophan, d-alanyl-d-alanine, and allantoate. Moreover, *Alloprevotella* exhibited a positive correlation with l-5-hydroxytryptophan, whereas *Megamonas* showed a positive correlation with spermine, l-histidinol, prostaglandin F2α, and nicotinic acid. Collectively, these three genera were negatively correlated with vitamin D_2_, ergocalciferol, and 2-methoxyresorcinol. In addition, *Enterococcus* and *Subdoligranulum*, which were increased in BD, were significantly and positively correlated with 4-aminobutyric acid. Moreover, the Eubacterium xylanophilum group and *Rhodococcus*, which were enriched in BD, were positively correlated with spermine and *trans*-zeatin, respectively. Conversely, *Blautia*, *Erysipelatoclostridium*, Clostridium innocuum group, *Paraprevotella*, *Fournierella*, *Akkermansia*, and *Lactobacillus*, which were decreased in BD, were positively correlated with vitamin D_2_, ergocalciferol, and 2-methoxyresorcinol. In addition, *Collinsella* and Ruminococcus gnavus group, which were decreased in BD, were positively correlated with docosapentaenoic acid, whereas *Solobacterium* was positively correlated with indole. Lastly, the decrease of *Oscillibacter* in BD was positively correlated with *N*-acetylhistamine, glycocholic acid, uric acid, 2-methoxyresorcinol, and *N*-(5-aminopentyl) acetamide and was negatively correlated with l-5-hydroxytryptophan, nicotinic acid, and d-alanyl-d-alanine.

## DISCUSSION

The present work consisted of a prospective case-control study to explore differences between healthy and acute diarrheic cats of two different breeds by the use of microbiome sequencing, nontargeted metabolomics, and metagenomic analysis. Although previous studies have investigated certain aspects of the relationship between the microbiome and acute diarrhea or IBD in cats (2, 13), the present study provided further insights into the relationship between the metabolome and microbiome in cats, as well as into the variations in their function.

The breed is a significant factor influencing the differentiation between health and disease states based on gut microbiome composition. Herein, *Firmicutes* and *Bacteroidota* were the most abundant phyla in the gut microbiota of American and British Shorthair cats; their ratio (*Firmicutes*/*Bacteroidota* [F/B]) decreased in cats with acute diarrhea, as shown previously for dogs with IBD (14). Notably, the baselines for the F/B ratio were significantly different in MH (1.30 ± 0.49) and BH (3.23 ± 2.82), which indicates that representatives of the phylum *Firmicutes* were more abundant in the gut microbiota of American Shorthair cats. Moreover, considering the functions predicted for the gut microbiota of cats evaluated herein, significant differences were found, with increased abundance of genes related to environmental information processing, such as membrane transport and signal transduction, in American Shorthair cats whereas energy metabolism was found increased in British Shorthair cats. Collectively, these findings suggest that the gut microbiota plays distinct roles and enables adaptive changes in the two cat breeds evaluated. This underscores the need to consider breed-specific differences and influences when investigating feline health and diarrhea states.

We identified a set of hallmark microbiota in cats with acute diarrhea that are shared between American and British Shorthair cats. Among them, we found that *Prevotella*, especially *P. copri*, is enriched in both cat breeds showing acute diarrhea, which was further validated by metagenomic analysis of British Shorthair cats. The role of intestinal *Prevotella* in human health is controversial. Numerous studies have shown an increase in *P. copri* in the gut of patients with type 2 diabetes (15), rheumatoid arthritis (16), and Parkinson’s disease (17). Herein, the increase in *P. copri* was closely associated with acute diarrhea in cats. In addition, it was shown that *Bacilli*, *Erysipelotrichales*, and *Erysipelatoclostridiaceae* were reduced in cats with acute diarrhea, which was similar to the findings of a previous study (13), thus possibly suggesting a role for these microorganisms in maintaining gut microbiota health in cats.

We noticed that downregulation of starch and sucrose metabolism was a common and distinctive feature of the gut microbiota of cats with acute diarrhea, although gut microbiota function varies by cat breeds. Moreover, a significant reduction in the abundance of genes related to glyceraldehyde dehydrogenase and pyruvate kinase was found in cats with acute diarrhea, based on metagenomic data. Glucose is degraded into pyruvate and ATP through the glycolytic pathway, and pyruvate is then utilized by microorganisms to synthesize important molecules, such as lactate and acetate, which maintain the pH of the GI tract and inhibit the growth of harmful bacteria. However, glyceraldehyde dehydrogenase and pyruvate kinase are important rate-limiting enzymes in the glycolytic pathway, and their reduction implies a decelerated glycolysis rate. Therefore, it can be suggested that the downregulation of starch and sucrose metabolism in cats with acute diarrhea is associated with a blockage of the glycolytic pathway, which thereby affects intestinal homeostasis. Moreover, 6-phospho-beta-glucosidase, beta-d-fucosidase, and abscisic acid (ABA)-specific beta-glucosidase were significantly reduced in cats with acute diarrhea, implying a reduction in the ability of intestinal microorganisms to utilize and degrade dietary cellulose (e.g., cellobiose) and to synthesize substances such as short-chain fatty acids (SCFAs).

In addition, pathways related to Vibrio cholerae infection, shigellosis, and the bacterial secretion system were enriched in BD, whereas pathways related to legionellosis and Salmonella infection were enriched in MD. Thus, these findings indicate that acute diarrhea in cats is closely associated with the colonization of the gut by potentially pathogenic bacteria. Moreover, lipopolysaccharide, an endotoxin, is a major component of the cell wall of Gram-negative bacteria. It has been demonstrated that upregulation of proteins and pathways related to lipopolysaccharide biosynthesis may indicate an increased multiplication rate of Gram-negative bacteria in the gut of cats with acute diarrhea.

We identified a group of intestinal metabolites that are closely related to acute diarrhea in cats, most of which are signal-regulating molecules represented by 4-aminobutyric acid, adenosine, and prostaglandin F2α. An increase in these metabolites was responsible for alterations in signaling pathways, including morphine addiction, neuroactive ligand-receptor interaction, phospholipase D signaling pathway, and GABAergic synapse in cats with acute diarrhea. Thus, we hypothesized that an increased abundance of 4-aminobutyric acid may be due to the stimulation of the stress response of gut microbes, which is closely related to *Enterococcus* and *Subdoligranulum*. Previous studies have shown increased concentrations of GABA in the fecal metabolome of dogs fed bone and raw food diets (18). Another study found that induction of enterotoxigenic Escherichia coli caused an increase in GABA concentration in the jejunum of piglets with diarrhea (19). Other studies described that ovalbumin caused an upregulation of intestinal GABAergic signaling in diarrheic mice and that blocking GABA signaling reduced the incidence of allergic diarrhea (20). In addition, a group of metabolites related to the sterol biosynthetic pathway (vitamin D_2_, ergocalciferol, and docosapentaenoic acid) was significantly reduced in the feces of cats with acute diarrhea, and the group of metabolites closely related to various bacteria (*Blautia*, *Akkermansia*, and *Lactobacillus*, etc.). These metabolites have been shown to exert anti-inflammatory and antioxidant effects in the gut (21, 22).

Tryptophan obtained through the diet is converted by intestinal bacteria into various indole metabolites which act as signaling molecules that contribute to intestinal immunity, thus exerting anti-inflammatory effects, inducing mucin expression, and enhancing intestinal mucosal resistance (23). In the present study, indole was significantly reduced in the feces of cats with acute diarrhea. An association between chronic enteropathy and an altered tryptophan-indole pathway has been suggested in dogs (24). Another important metabolite of tryptophan, l-5-hydroxytryptophan, was found in significantly higher levels in the feces of diarrheic cats. Moreover, l-5-hydroxytryptophan, a precursor of 5-hydroxytryptamine, may lead to an increase in the availability of 5-hydroxytryptamine in the gut, thus leading to diarrhea, since the release of this precursor has been associated with watery diarrhea in ulcerative colitis and postprandial dyspepsia (25, 26). Remarkably, *Rhodococcus* was significantly increased in the gut of cats with acute diarrhea and showed a significant positive correlation with *trans*-zeatin. It has been shown that Rhodococcus fascians depended on a linear plasmid-encoded Fas manipulator to produce *trans*-zeatin, which is cytotoxic to plant cells (27, 28). In contrast, here, a similar pattern in animals has been shown for the first time as a possible pathway leading to diarrhea in cats.

Furthermore, among the advantages of the present study, the following can be cited: first, the study cohort included multicentric samples of two cat breeds, thus avoiding the bias of a single environmental factor and a single breed; second, we used multiomics validation to better explain the relationship between changes in the gut microbiome and metabolites of cats; and third, covariates for confounding factors, such as age and gender, were corrected, thus demonstrating that breed and disease are important factors influencing the gut microbiome of cats. In particular, our predictive model for diagnosing acute diarrhea in cats yielded high AUCs. Furthermore, Tax4Fun, an R package for functional prediction based on the 16S rRNA SILVA database was used, which has been shown to be superior to PICRUSt functional prediction in terms of predictive accuracy (29).

Similar to what some articles have reported in this field (11, 30), this research possesses certain limitations. First, the sample scale is relatively small, which restricts our ability to generalize the findings to other phenotypes (diseases and breeds) and a more diverse cat population. Second, due to the limited sample size and the current lack of available data on the gut microbiome of cats with acute diarrhea, our diagnostic model has not undergone external validation, i.e., applying the model to an independent data set to assess its generalizability and stability. Third, our study did not further elucidate the causal relationship between gut microbiota, metabolites, and acute diarrhea. However, it has been demonstrated that abnormal fecal metabolites can influence the gut microbiota and host health through the brain-gut axis and gut-liver axis (31). Taken together, the results presented here provide important clues and foundational data accumulation for a better understanding of GI diseases in cats.

In summary, we characterized the microbiomes and metabolomes from two different breeds of cats, recognizing a class of intestinal flora and metabolites in cats with acute diarrhea. These metabolites were involved in three significantly altered metabolic pathways, i.e., steroid biosynthesis, morphine addiction, and neuroactive ligand-receptor interactions, and were closely associated with certain differential genera. On this basis, factors that could be strongly associated with acute diarrhea in cats, which included reduced carbohydrate metabolism, reduced ability to produce SCFAs from fiber, abnormal tryptophan metabolism, multiplication of potentially pathogenic bacteria, and inflammatory responses to multiple signaling molecular pathways, were unveiled. Collectively, we identified differential flora and metabolites in feline acute diarrhea states and established a diagnostic model by screening out relevant microbial taxa. These findings provide valuable insights for subtyping determination and targeted treatment of gastrointestinal diseases in cats. However, further research with larger cohorts of cats and diverse conditions is needed to validate and refine the diagnostic model. This will contribute to a better understanding of feline gastrointestinal health and improve disease management.

## MATERIALS AND METHODS

### Animals and sample collection.

This study conducted a case-control prospective cohort study. Healthy cats and cats with acute diarrhea of two different breeds, American Shorthair and British Shorthair, were recruited from various regions in China. Accordingly, based on their physiological status (healthy or diarrheal) and breed, they were assigned to four main sample groups, as follows: healthy American Shorthair (MH), healthy British Shorthair (BH), diarrheic American Shorthair (MD), and diarrheic British Shorthair (BD). Moreover, animals were classified as normal or obese based on the Purina body condition assessment criteria (32). All cats included in the study originated from private owners and were fed a variety of commercial diets. Criteria for determining healthy or disease state were based on detailed clinical evaluation provided by cat owners (i.e., feeding habits, exercise, vomiting, body condition score, fecal score, disease history, and medication/antibiotic/probiotic/prebiotic use, among others). Clinical information on the recruited cats is provided in Table S1 in the supplemental material. Fecal scoring criteria were divided into seven categories: 1, granular; 2, sausage-like but with surface clumps; 3, sausage-like but with cracks on the surface; 4, snake-like with a smooth surface; 5, marshmallow-like with clear edges and semisolid consistency; 6, porridge-like without a regular shape; and 7, liquid without solid clumps (33). Healthy cats enrolled in the study met the following criteria: fecal score of 1 to 4 and no history of GI disease for at least 6 months before and 3 weeks after stool sampling. Cats with acute diarrhea were enrolled in the study if they met the following inclusion criteria: fecal score of 5 to 7 and presenting with diarrhea within 2 weeks before and after sample collection. Exclusion criteria encompassed the following: use of antibiotics, probiotics, prebiotics, or other medications within 1 month prior to fecal sampling; history of vaccination and internal parasite treatment within the 6 months prior to fecal sampling; current presence of parasitic or viral infections; and occurrence of acute or idiopathic non-GI diseases within 6 months prior to fecal sampling, including but not limited to inflammatory conditions (such as respiratory, endocrine, and urinary tract inflammations), trauma, as well as other medical and surgical diseases; and presence of systemic and chronic diseases, including but not limited to diabetes, systemic lupus erythematosus, arthritis, and tumors. The fecal samples, obtained by the owners from the cats after voluntary defecation, were collected into cryovials. Subsequently, the cryovials were placed in a portable cooler set at −20°C for frozen transportation to the laboratory. Upon arrival, the samples were stored at −80°C for long-term preservation. All experiments were conducted based on fecal samples. Written informed consent was obtained from all cat owners. No specific ethical approval was required for conducting the present experiments since fecal samples evaluated herein were obtained after voluntary excretion by cats.

### 16S rRNA gene sequencing.

Genomic DNA from all fecal samples was extracted using the cetyltrimethylammonium bromide method. The concentration of extracted DNA was determined by agarose gel electrophoresis. As a negative control, ultrapure water was processed alongside the fecal samples using the same extraction reagents and procedures. Subsequently, the V4 hypervariable region of the bacterial 16S rRNA gene was amplified using specific primers (515F and 806R) and Phusion high-fidelity PCR master mix with GC buffer (New England Biolabs, Ipswich, MA, USA). PCR products were subjected to library construction with a NEBNext Ultra II DNA library prep kit (New England Biolabs), followed by sequencing on an Illumina NovaSeq 6000 platform. Sequencing data were submitted to barcode splitting, followed by splicing of reads to obtain raw sequences using Flash software (v.1.2.11; http://ccb.jhu.edu/software/FLASH/). Raw sequences were screened using fastp software, and chimeras were removed using Vsearch software. Sequences whose abundance was lower than 5 were removed to obtain final amplicon sequence variants (ASVs) using the QIIME2 DADA2 pipeline. Subsequently, the ASVs obtained were compared with the SILVA database (v. 138.1) for species annotation, using the QIIME2 classify-sklearn pipeline.

### 16S rRNA gene sequencing information analysis.

**(i) Diversity analysis.** Alpha-diversity indexes were calculated using the QIIME2 pipeline to assess microbial community diversity within samples. Among these, observed OTU, Chao1, and dominance indexes were used to evaluate community richness; Shannon and Simpson indexes were used to evaluate community diversity; the Pielou index was used to reflect species evenness. Significant differences between groups were analyzed using the Wilcoxon rank sum test. In addition, principal-coordinate analysis (PCoA) was performed based on the weighted UniFrac distance, and the principal coordinate with the largest contribution rate was selected for plotting using the ggplot2 package in R software (version 2.15.3). Statistical significance of sample groups was determined by permutation multivariate analysis of variance (Adonis) using the R vegan package.

**(ii) Analysis of species difference.** Multivariate analysis by linear models (MaAsLin) was performed to correct the effect on microbial community abundance in different groups, using gender, age, species, or disease as covariates (https://huttenhower.sph.harvard.edu/galaxy/). The following criteria were used to filter data: significance threshold, 0.05; minimum feature relative abundance, 0.0001; and minimum feature prevalence, 0.01. Subsequently, linear discriminant analysis (LDA) with effect size (LEfSe) was conducted to identify bacterial taxa differentially enriched among sample groups. Features with significantly different abundances between sample groups were screened and investigated using the Wilcoxon rank sum test and the LEfSe package (version 1.0). LDA was conducted, and LDA scores of >4 were considered significantly differentially enriched features. Metastats software (http://metastats.cbcb.umd.edu/) was used to obtain *P* values from hypothesis tests comparing species abundances between sample groups at the phylum and genus level. In addition, *t* permutation analysis for widely distributed bacteria and Fisher’s exact test were conducted for sparsely distributed bacteria.

**(iii) Tax4Fun function prediction.** The function of obtained 16S rRNA gene sequencing was predicted using the Tax4Fun R package (29). Specifically, obtained sequences were clustered and annotated with functional information at three annotation levels, using the 16S SILVA database as reference. The top 35 functions with the largest abundance at each annotation level were selected and used to construct a heat map and clustered based on the level of functional difference between different groups as obtained with the *t* test.

**(iv) Random forest models.** In addition, to establish feature prediction models to distinguish cats with acute diarrhea from healthy cats, random forest models were constructed for 24 diarrheic cats and 24 healthy cats using the Random Forest R package (version 3.2.1). First, ASV data were corrected for age, gender, and breed for more accurate predictions. Subsequently, species were selected based on gradient (genus level), and significant species were screened by supervising mean decrease accuracy and mean decrease gin indexes, followed by 10-fold cross-validation and plotting of the ROC curve to obtain the classification prediction model with the best sensitivity and specificity. Finally, to determine microbial cooccurrence relationships in cats with acute diarrhea, species Spearman correlation coefficients for all samples were calculated using the R package (version 3.4.0) and filtered to remove connections with correlation coefficients of <0.6, node self-connections, and node abundances of <0.005%. Finally, cooccurrence network plots were obtained using graphviz software (version 2.38.0).

### Metagenome sequencing and analysis.

Metagenome sequencing was performed on four representative samples each from healthy and diarrheic British Shorthair cats. Genomic DNA was extracted using a Tiangen magnetic bead kit, and as a negative control, ultrapure water was processed alongside the samples using the same extraction reagents and procedures. The extracted DNA was then subjected to random fragmentation, end repair, poly(A) tail length control, and sequencing junction addition. PCR amplifications were then conducted to complete the library preparation, followed by sequencing on an Illumina PE150 platform. Low-quality reads were called from raw sequencing data using the Readfq package (version 8; https://github.com/cjfields/readfq) and filtered using Bowtie2 software (version 2.2.4; http://bowtie-bio.sourceforge.net/bowtie2/index.shtml) to obtain clean sequencing data. Sequencing data were then assembled to obtain scaftigs for each sample using MEGAHIT software (v1.0.4-beta) (34). Scaftigs were subjected to gene prediction using MetaGeneMark (http://exon.gatech.edu/GeneMark/) to construct a gene catalogue, which was subsequently compared with the MicroNR library for obtaining species annotation information for each gene (UniGenes). Finally, species abundance of each sample at different taxonomic levels was calculated. UniGenes were matched against functional databases to obtain functional annotations and functional abundance for each sample using Diamond software (v.0.9.9.110; http://bowtie-bio.sourceforge.net/bowtie2/index.shtml). Functional databases included in the present study were the Kyoto Encyclopedia of Genes and Genomes (KEGG) (version 20180101; http://www.kegg.jp/kegg/), the Evolutionary Genealogy of Genes: Nonsupervised Orthologous Groups (eggNOG) (version 4.5; http://eggnogdb.embl.de/#/app/home), and the carbohydrate-active enzyme (CAZy) (version 201801; http://www.cazy.org/) databases.

Similar to 16S rRNA gene sequencing analysis, grouped differential species were screened using LEfSe. Based on the relative abundance of functions at different taxonomic levels, functions with significant differences between groups were determined using Metastats software.

### Nontargeted metabolomic analysis.

Nontargeted metabolomic analysis of fecal samples from eight healthy and eight diarrheic representatives of British Shorthair cats was performed by Novogene Co., Ltd. (Tianjin, China). All samples were analyzed by liquid chromatography-mass spectrometry for microbial metabolite quantification in fecal samples. Sample preparation procedure, experimental parameters, raw data pretreatment, and metabolite annotation pipeline are provided in the supplemental material.

Metabolomic data analysis was performed using R (version R-3.4.3) and Python (version 3.5.0) software. To determine biological significance of groups, partial least-squares discriminant analysis (PLS-DA) was performed using MetaX software (35), and the variable importance in projection (VIP) values of the first principal component were calculated. Fold change (FC) values were calculated as the ratio of the means of quantitative values of all biological replicates for each metabolite within each comparison group. *t* tests were conducted to determine the significance of each metabolite between comparison groups and obtain *P* values. Significantly differential metabolites between BH and BD subgroups were considered based on the following criteria: VIP value of >1, *P* value of <0.05, and FC value of ≥2 or <0.5.

Moreover, a hierarchical cluster analysis was performed on all differential metabolites to determine differences in the distribution of metabolite profiles between experimental groups. The relative quantitative values of differential metabolites were normalized using the z-score, and metabolites with the same or similar metabolic profiles were clustered using the Pheatmap package in R to create a heat map (36). In addition, to investigate the main biological functions exercised by differential metabolites, KEGG pathway enrichment analysis was conducted using the KEGG database (https://www.genome.jp/kegg/pathway.html). Based on the results of metabolite annotation using the KEGG database, the number of metabolites involved in a given metabolic pathway for all metabolites was defined as *N*, the number of differential metabolites in *N* was defined as *n*, the number of metabolites annotated to a particular KEGG pathway was defined as *y*, and the number of differential metabolites enriched within a particular KEGG pathway was defined as *x*. If the ratio condition *x*/*n* > *y*/*N* was satisfied, then the pathway was considered an enriched pathway. Enriched pathways were then tested using a hypergeometric test, and *P* values of ≤0.05 were considered to define a KEGG pathway as significantly enriched in differential metabolites. Bubble plots of enriched pathways were plotted using the R package ggplot2. To investigate the degree of association between species diversity and metabolites in samples, 23 differential genera and 20 significantly differential metabolites were selected between BH and BD sample groups for Pearson’s correlation analysis. Pearson’s correlation coefficients (rho) and *P* values for differential genera and metabolites were calculated using the correlation analysis module of the NovoMagic platform from Novogene, and the results showing the criteria |rho| ≥ 0.8 and *P *≤ 0.05 were selected for scatter plotting.

### Statistical analysis.

The age, body weight, fecal score, duration of diarrhea, defecation frequency, and dietary nutrient content (protein, fat, and carbohydrates) of cats in different experimental groups are presented as the mean ± SD. Statistical significance between different experimental groups was determined using the Kruskal-Wallis test for continuous variables and Fisher’s exact test for categorical variables.

### Data availability.

Data are available in public repositories which are open access. The 16S amplicon sequencing data generated and analyzed in this study have been deposited in the Sequence Read Archive of the NCBI database under accession no. PRJNA908214. The metagenome sequencing data have been deposited in the Sequence Read Archive under accession no. PRJNA908260. Nontarget metabolome data can be obtained from the corresponding author. Additional data generated or analyzed during this study are included in this published article (and its supplemental material files).
