# Supplementary material for: Identification of Gut Microbiome and Metabolites Associated with Acute Diarrhea in Cats
Source: Microbiol Spectr. 2023 Jul 10;11(4):e00590-23. doi: 10.1128/spectrum.00590-23 (PMC10434016; doi:10.1128/spectrum.00590-23)
Supplement: Supplemental file 6 — Supplemental material. Download spectrum.00590-23-s0001.docx, DOCX file, 9.1 MB [file spectrum.00590-23-s0001.docx]

**Supplementary Material**

**Identification of gut microbiome and metabolites associated with acute diarrhea in cats**

Huasong Bai^a^, Tong Liu^a^, Songjun Wang^a^, Wenhui Gong^a^, Liya Shen^a^, Song Zhang^a^, Zhanzhong Wang^a^#

*^a^Nourse* *Science Centre for Pet Nutrition, Wuhu 241200, China*

^*^Corresponding author: [wzz7698@tju.edu.cn](mailto:wzz7698@tju.edu.cn)

# *1. Fecal untargeted metabolomics*

100 mg of fecal samples ground in liquid nitrogen were added to 500 μL of 80% methanol aqueous solution, vortexed, placed in an ice bath for 5 min, and centrifuged at 15,000 g at 4 °C for 20 min. A certain amount of supernatant was diluted with mass spectrometry-grade water to a methanol content of 53%, centrifuged at 15,000 g at 4 °C for 20 min, and the supernatant was collected and injected into liquid chromatography-mass spectrometry (LC-MS) for analysis ^1^. All samples were analyzed with a liquid chromatography instrument (Vanquish UHPLC, Thermo Fisher, Germany) and a time-of-flight mass spectrometer (Q Exactive HF-X, Thermo Fisher, Germany). The chromatographic conditions were as follows: the column was Hypesil Gold column (C18, Thermo Fisher USA); the column temperature was 40 °C; the flow rate was 0.2 mL/min; the mobile phase in positive ion mode was 0.1% formic acid (A117-50, Thermo Fisher, USA) and methanol (A456-4, Thermo Fisher, USA); the mobile phase in negative ion mode was 5 mM Ammonium acetate (pH 9.0, A114-50, Thermo Fisher, USA) and methanol. Mass spectrometry was performed in positive and negative ion mode, and the scan range was selected from m/z 100-1500. The settings of the ESI source were as follows: spray voltage 3.5 kV; sheath gas flow rate 35 psi; auxiliary gas flow rate 10 L/min; ion transfer tube temperature 320 °C; iontophoresis RF level 60; Auxiliary gas heater temperature 350 °C. The unloaded raw data was preprocessed with CD 3.1 library search software (Novogene) for peak alignment, peak extraction and relative quantification. The retention time was set to 0.2 min, the signal deviation was 5 ppm, the signal intensity deviation was 30%, and the signal-to-noise ratio was 3. Metabolomic data analysis was performed based on the software R (version R-3.4.3) and Python (version 3.5.0). The identified metabolites were annotated using the KEGG database (<https://www.genome.jp/kegg/pathway.html>), the HMDB database (<https://hmdb.ca/metabolites>) and the LIPIDMaps database (<http://www.lipidmaps.org/>).

# *2. Results*


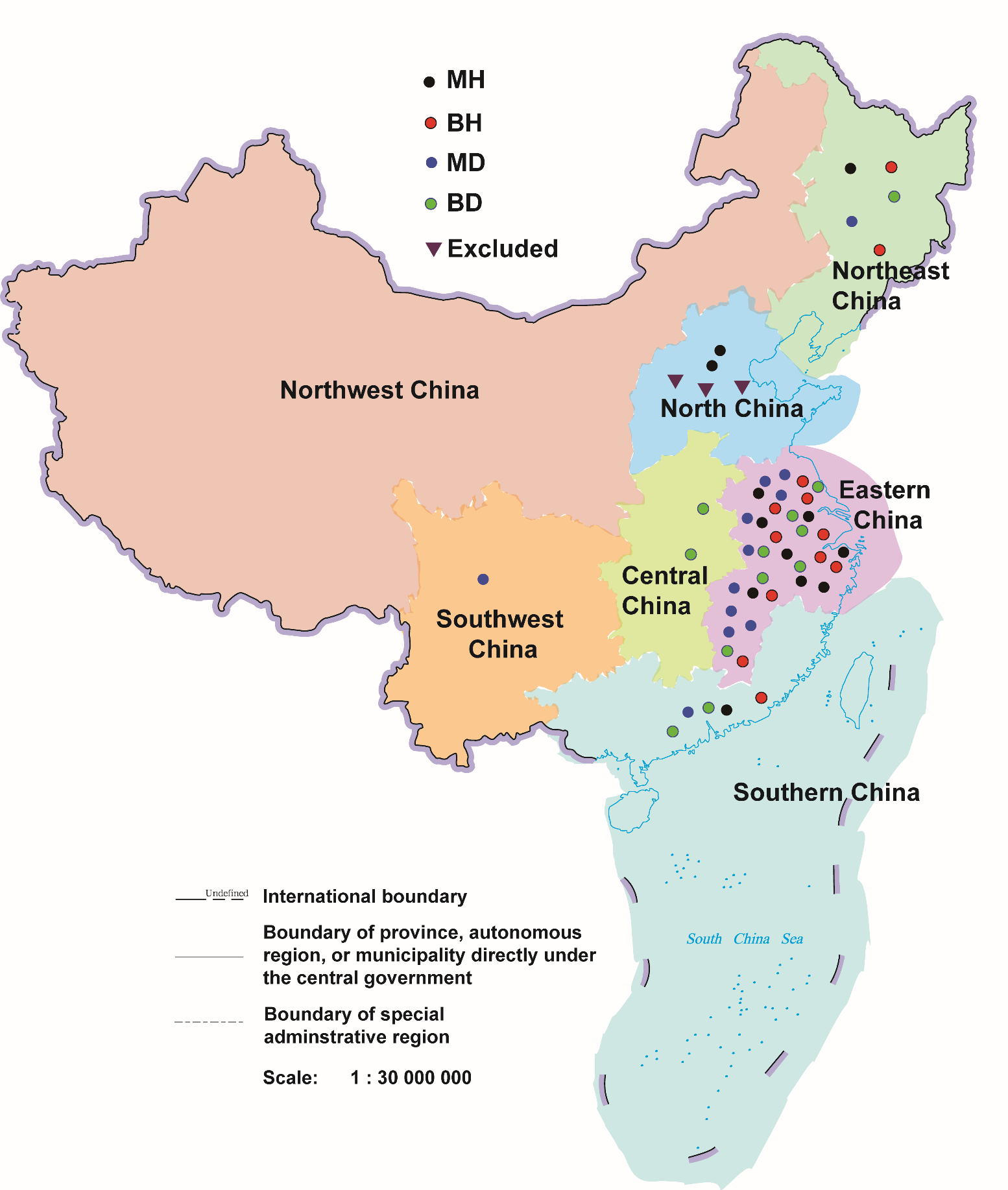


**Figure S1. Schematic map depicting the geographic locations of animals recruited from different regions in China.** The four different groups are represented by distinct colored circular symbols: MH (Healthy American Shorthair), MD (American Shorthair with acute diarrhea), BH (Healthy British Shorthair), and BD (British Shorthair with acute diarrhea). The triangle symbols indicate animals that were excluded from the study due to not meeting the inclusion criteria. The scale of the map is 1:30,000,000.


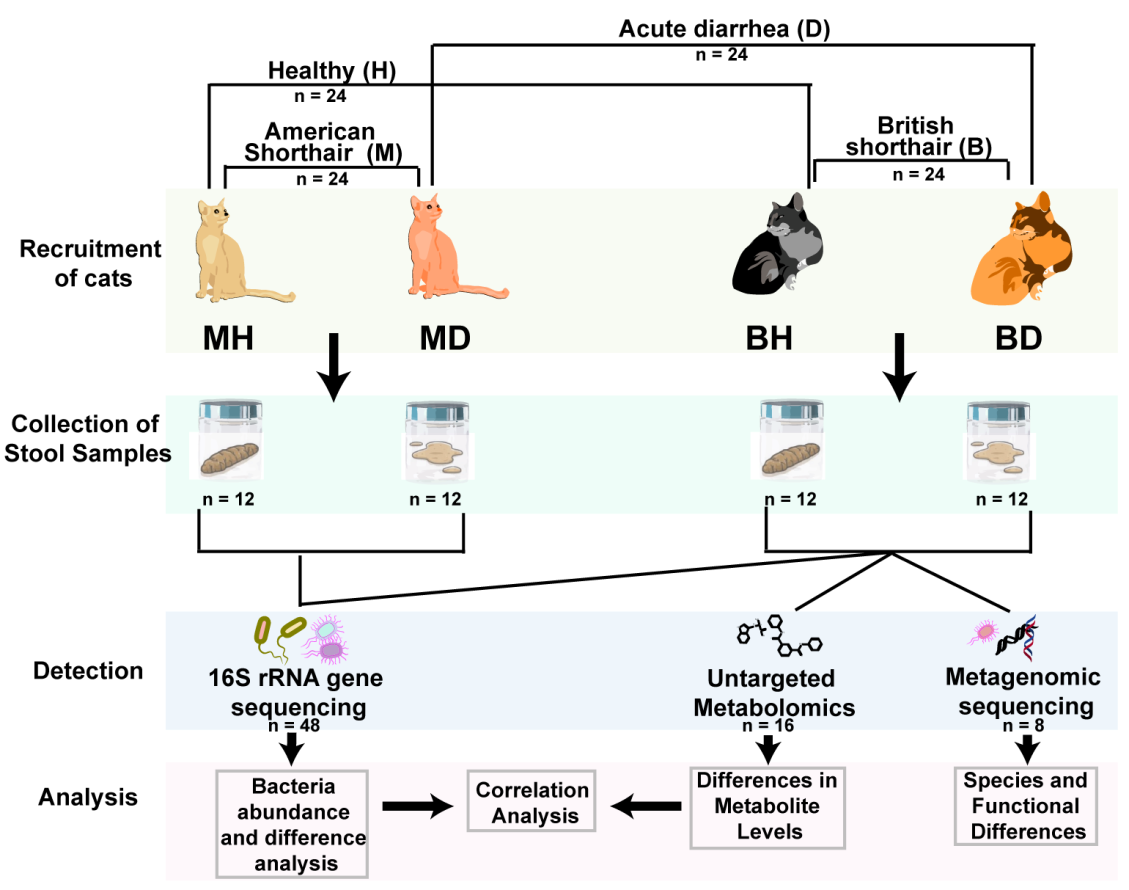


**Figure S2. Overview of the study design.** A total of 48 cats, 12 American Shorthairs with acute diarrhoea, 12 British Shorthairs with acute diarrhoea, 12 healthy American Shorthairs and 12 healthy British Shorthairs, were recruited from 6 regions in China and sequenced for intestinal faecal microbial 16S rRNA. Metagenomic sequencing (n = 8) and non-targeted metabolomic analysis (n = 16) were also performed on the faeces of British Shorthairs.


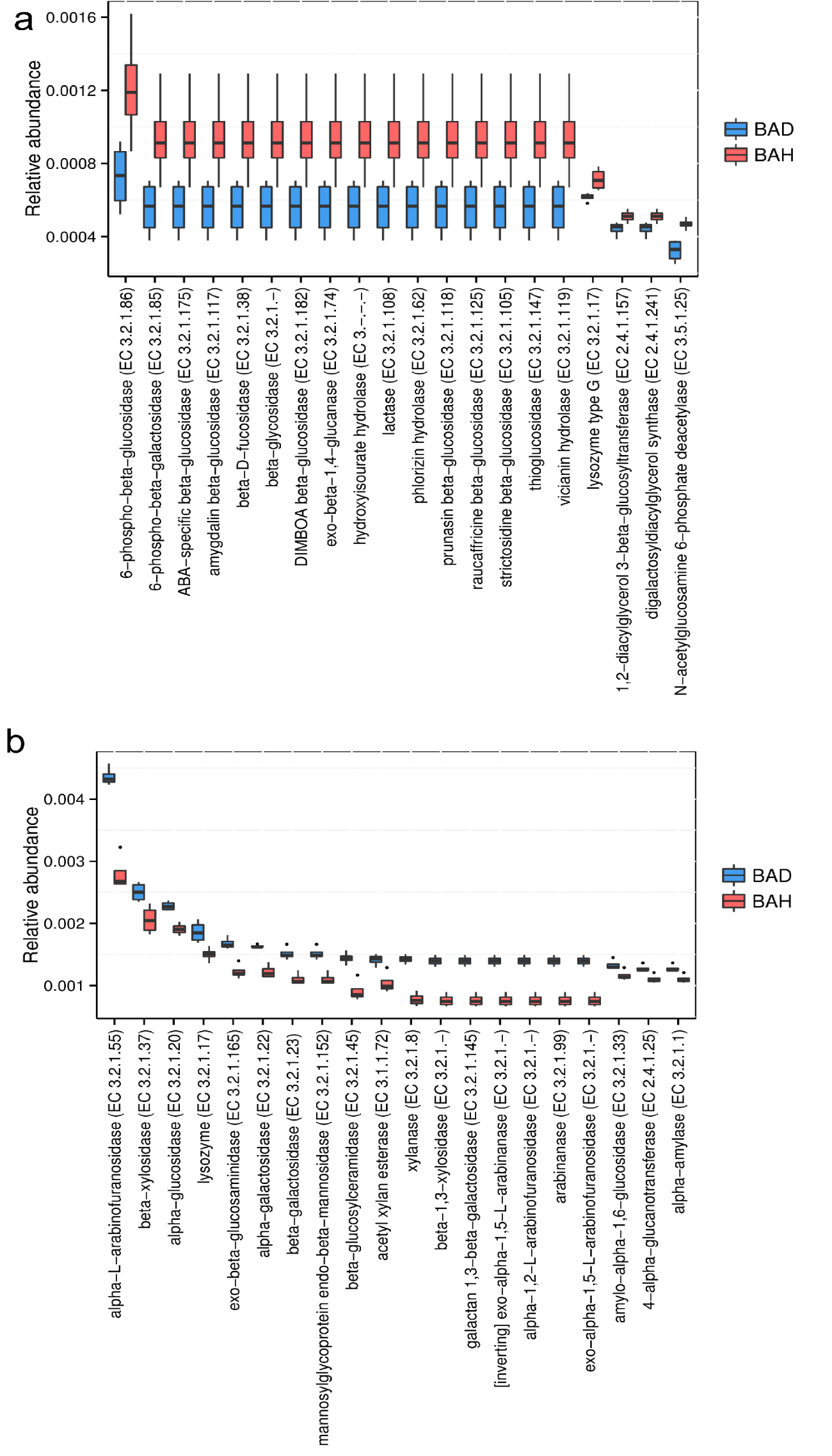


**Figure S3.** **Box plot of relative functional abundance with significant differences between healthy American shorthairs (BAH) and and acute diarrhea American shorthairs (BAD), based on functional annotations from macrogenome sequencing in the CAZy database.** (a) reduced CAZy functions in BAD. (b) elevated CAZy functions in BAD. All functions demonstrated had significance *P* values less than 0.05 between the two groups (Metastats analysis).

**
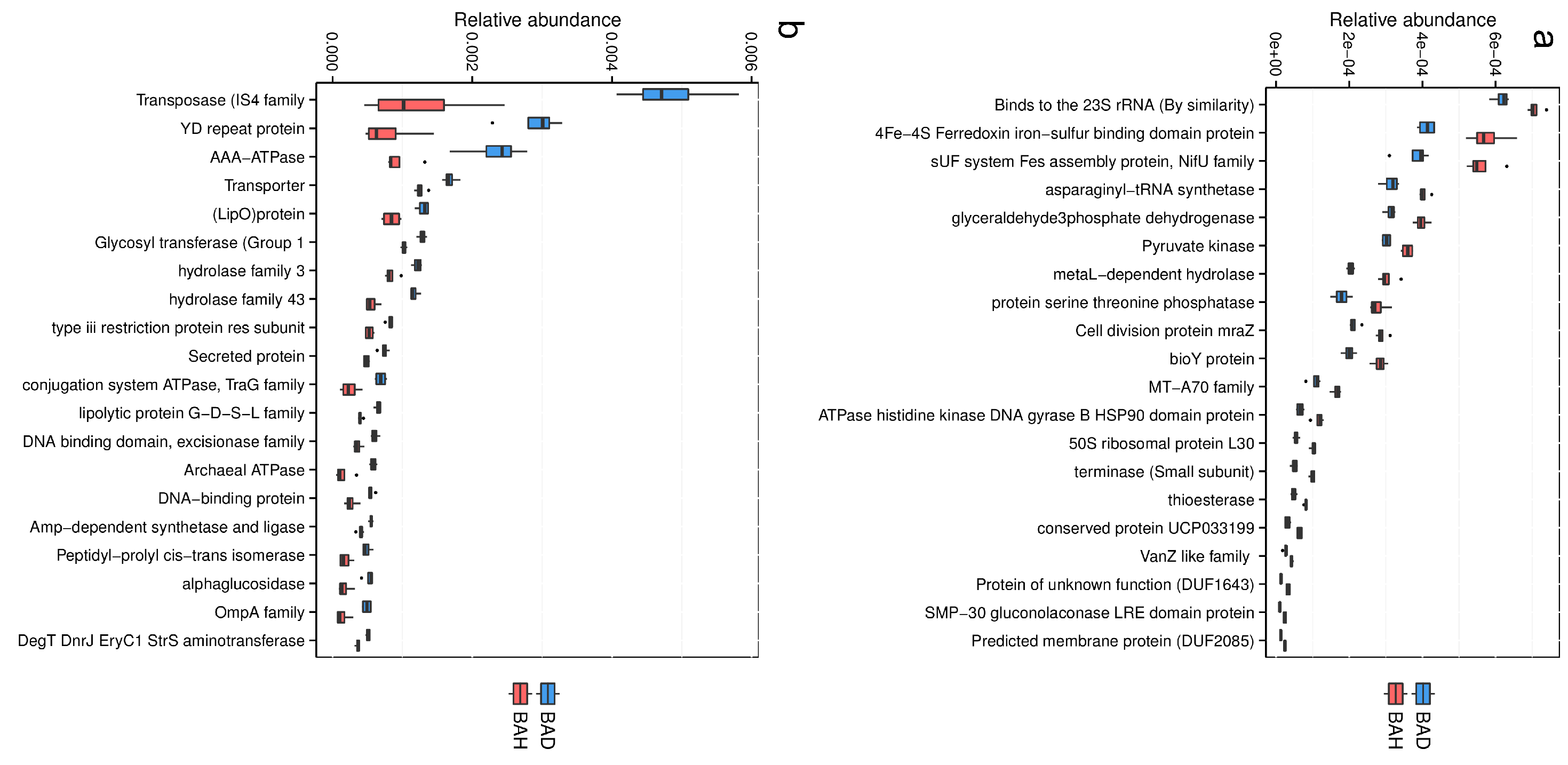
**

**Figure S4.** **Box plot of relative functional abundance with significant differences between healthy American shorthairs (BAH) and and acute diarrhea American shorthairs (BAD), based on functional annotations from macrogenome sequencing in the eggNOG database.** (a) reduced orthologous groups in BAD. (b) elevated orthologous groups in BAD. All functions demonstrated had significance *P* values less than 0.05 between the two groups (Metastats analysis).

**
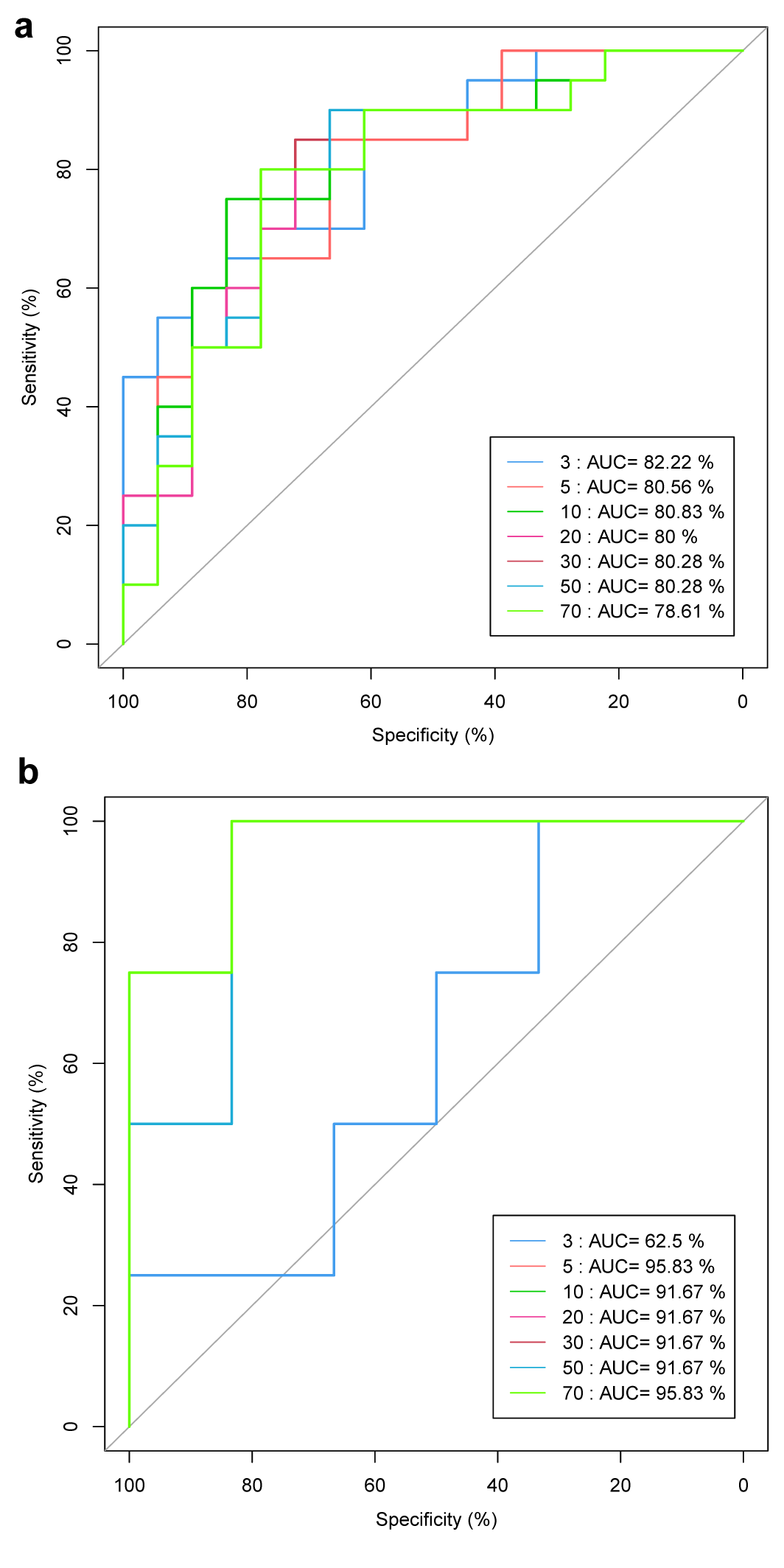
**

**Figure S5. Receiver operating characteristic curves (ROC) of random forest model with 7 splits for the construction of a diagnostic classifier for acute diarrhoea in cats.** (a) ROC of the random forest model for 7 draws in the training set. (b) ROC of the random forest model in the test set for the 7-sample validation. AUC is the area of the curve under the ROC.

References

1. Want EJ, Masson P, Michopoulos F, Wilson ID, Theodoridis G, Plumb RS, Shockcor J, Loftus N, Holmes E, Nicholson JK. Global metabolic profiling of animal and human tissues via UPLC-MS. Nat Protoc. 2013; 8:17-32.
